# A time-course transcriptome analysis of gonads from yellow catfish (*Pelteobagrus fulvidraco*) reveals genes associated with gonad development

**DOI:** 10.1186/s12864-022-08651-0

**Published:** 2022-05-31

**Authors:** Dong Gao, Junrou Huang, Genmei Lin, Jianguo Lu

**Affiliations:** 1 School of Marine Sciences, Sun Yat-Sen University, Zhuhai, 519082 China; 2 Southern Marine Science and Engineering Guangdong Laboratory (Zhuhai), Zhuhai, 519080 China; 3 Guangdong Provincial Key Laboratory of Marine Resources and Coastal Engineering, Guangzhou, 510275 Guangdong China; 4 Pearl River Estuary Marine Ecosystem Research Station, Ministry of Education, Zhuhai, 519000 China

**Keywords:** Time-course transcriptome, Sexual dimorphism, Gonad development

## Abstract

**Background:**

The yellow catfish, *Pelteobagrus fulvidraco*, is a commercially important fish species. It is widely distributed in the fresh water areas of China, including rivers, lakes, and reservoirs. Like many other aquaculture fish species, people have observed significant size dimorphism between male and female yellow catfish and it shows a growth advantage in males.

**Results:**

Here, at the first time, the time-course transcriptome was used to explore the various expression profiles of genes in different gonad developmental stages and genders. A total of 2696 different expression genes (DEGs) were identified from different stages. Based on these DEGs, 13 gonad development related genes were identified which showed time-specific or sex biased expression patterns.

**Conclusion:**

This study will provide the crucial information on the molecular mechanism of gonad development of female and male yellow catfish. Especially, during the different gonad development stages, these 13 gonad development related genes exhibit various expression patterns in female and male individual respectively. These results could inspire and facilitate us to understanding the various roles of these genes play in different gonad development stages and genders.

**Supplementary Information:**

The online version contains supplementary material available at 10.1186/s12864-022-08651-0.

## Background

Yellow catfish, *Pelteobagrus fulvidraco*, has become an important freshwater variety in Chinese aquaculture (cite China Fishery Statistical Yearbook 2018). Because of its tender flesh, few inter-muscular spines, and delicious flavor, this fish becomes much more popular in China. Meanwhile, yellow catfish also has been regarded as an ideal model to research the sexual dimorphism, considering the significant different growth rates and maximum body sizes between female and male yellow catfish [1]. So, the studies of genetic mechanisms of sex dimorphism are crucial to yellow catfish. It is also benefited for yellow catfish aquaculture.

Gonad is the primary organ presenting morphological signs of sexual dimorphism. Thus, the studies of gonad development genes could provide valuable insights in studying sexual dimorphism [2]. To date, many gonad development genes have been identified for several fish species, including *dmrt1* (double-sex and mab-3 related transcription factor 1) in half-tongue sole (*Cynoglossus semilaevis*), *sdy* (sexually dimorphic on the Y chromosome) in rainbow trout (*Oncorhynchus mykiss*), and *amhr2* (anti-Muellerian hormone receptor type II) in tiger pufferfish (*Takifugu rubripes*) [3].

For yellow catfish, although several gonad development genes have been detected by previous studies, such as *amhr2*, *gdf9* (growth and differentiation factor 9), *cyp1a1* (cytochrome P450 1A1), *cyp19a* (cytochrome P450 19A), *piwi* (P-element induced wimpy testis like) and *vasa* [4–6]. The performance of different gonad development genes in various gonad development stages is still ambiguous for yellow catfish.

In order to obtain the various expression patterns of gonad development genes during the entire gonad development stages of yellow catfish. A time-course gonadal transcriptome of yellow catfish was generated with high-through sequencing. The DEGs were identified among different comparison groups. The further annotation of gonad development DEGs may help us to illuminate the mechanism involved in gonad development and sexual dimorphism.

## Results

### Overview of RNA-Seq results

**Fig. 1:**
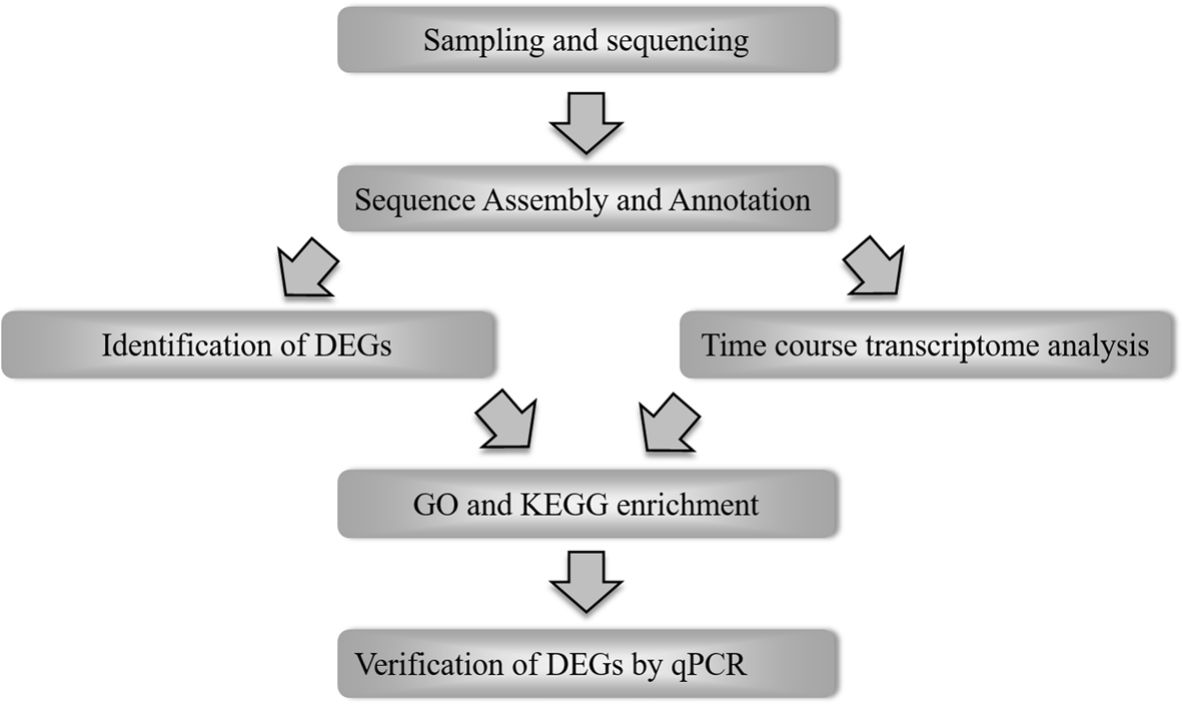
Flowchart of the pipeline used to a time-course transcriptome analysis of gonads from yellow catfish

In order to identify gonad development related genes in yellow catfish, the RNA-Seq was performed in all of sample groups. The data sets yielded from 8.54 Gbp to 16.38 Gbp and the average mapping rate for *P. fulvidraco* reference genome was 84.24%. The flowchart of processing steps in our pipeline is shown in Fig. 1. The sequencing results were listed in Table 1.

**Table 1:** Summary of the RNA-Seq data sets and the mapping ratios for all sample groups. Note: The sample names represent the different gonad development stages when the samples were collected. BG: before gonad differentiation; MG: male gonad differentiation; MGC: male gonad differentiation control; FG: female gonad differentiation; FGC: female gonad differentiation control; MAG: male after gonad differentiation; FAG: female after gonad differentiation

Sample name	Raw data (Gbp)	Clean data (Gbp)	Map rate (%)
BG	16.38	14.50	84.4
MG	10.46	9.26	83.8
MGC	8.54	7.56	83.1
FG	9.12	8.08	84.3
FGC	8.62	7.64	85.0
MAG	13.68	12.12	84.1
FAG	11.74	10.40	85.0
Total	78.54	69.56	–

### Transcriptome assembly, annotation and Avaluation

Based on mapped reads, the time-coure transcriptome of *P. fulvidraco* was generated by Cufflinks. A total size of draft transcriptome was 271.25 Mbp including over 85,880 raw transcripts. In order to obtain a final assembly, two filter metrics were set, including the minor length is 150 bp and this transcript must express (FPKM > 0) at least in a group. The final assembly consisted of 41,329 transcripts. These transcripts were annotated via searching against the Nr, GO and KEGG database respectively. There were 34,273 transcripts corresponding to 16,861 genes, with an E-value cutoff less than 1e-5. A total number of 29,303 transcripts were assigned at least one GO term, corresponding to 14,637 genes. 14,223 transcripts were annotated into 344 KEGG pathways (Additional file 1). The Benchmarking Universal Single-Copy Orthologs (BUSCO) was used to estimate transcriptome completeness using the actinopterygii_odb9 BUSCO set [7]. Among all of (4584) BUSCO core genes, 3694 (80.6%) BUSCO core genes were identified in the yellow catfish transcriptome which suggested that this assembly was good enough to subsequent analysis.

### Identification of gonad development related DEGs

**Fig. 2:**
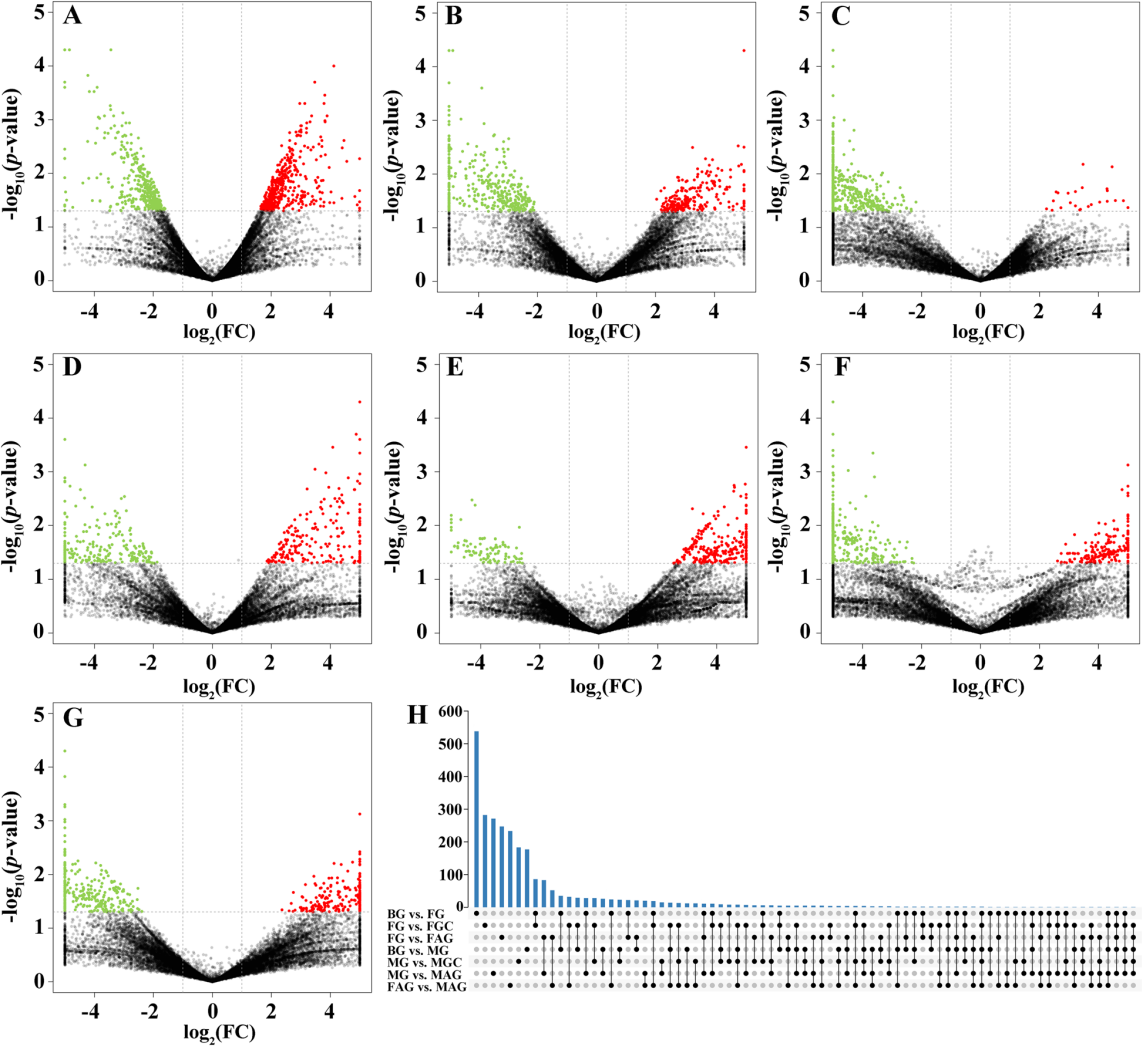
These volcano plots displayed the number of differentially expressed genes between the comparison libraries. **A** BG vs. FG comparison group; (**B**) FG vs. FGC comparison group; (**C**) FG vs. FAG comparison group; (**D**) BG vs. MG comparison group; (**E**) MG vs. MGC comparison group; (**F**) MG vs. MAG comparison group; (**G**) FAG vs. MAG comparison group; (**H**) a Venn diagram of all comparison groups

According to the method, 2696 DEGs were identified from all of these comparison groups. For the different ovary development stages, several gonad development related DEGs were detected in Fig. 2A-C and Table 2. For example, for BG vs. FG, 447 DEGs were up-regulated including *Aurka* (aurora kinase A), *cyp1a1*, *piwi*, *plk1* (polo-like kinase 1) and *vasa*. For FG vs. FGC, *Aurka*, *cyp1a1*, *org* (oogenesis-related isoform A), *piwi*, *plk1* and *vasa* were significantly highly expressed in female individual (FG). *Cyp1a1* was down-regulated in FG vs. FAG comparison group.

**Table 2:** Gonad development related DEGs in yellow catfish

Gene name	Description	Gene id	Comparison groups						
			BG vs. FG	FG vs. FGC	FG vs. FAG	BG vs. MG	MG vs. MGC	MG vs. MAG	FAG vs. MAG
*amhr2*	anti-Muellerian hormone type-2 receptor	XLOC_006098						↑	
*aurka*	aurora kinase A	XLOC_019110	↑	↓			↑		
*bmp15*	bone morphogenetic protein 15	XLOC_000571							↓
*cyp1a1*	cytochrome P450 1A1	XLOC_007208	↑	↓	↓	↑		↓	
*gdf9*	growth/differentiation factor 9-like	XLOC_003614							↓
*org*	oogenesis-related isoform A	XLOC_016409		↓					↓
*piwi*	piwi-like protein	XLOC_008581	↑	↓				↑	
*plk1*	polo-like kinase 1	XLOC_013011	↑	↓					
*sox9*	transcription factor Sox-9-A-like	XLOC_010029							↑
*spata22*	spermatogenesis-associated protein 22	XLOC_024282						↑	
*tex11*	testis-expressed sequence 11 protein	XLOC_012328						↑	
*tex15*	testis-expressed sequence 15 protein	XLOC_018159						↑	
*vasa*	ATP-dependent RNA helicase DDX4	XLOC_017737	↑	↓				↑	

For the different testis development stages, *cyp1a1* was up-regulated in BG vs. MG. In the MG vs. MAG comparison group, *amhr2*, *piwi* and *vasa* showed higher expressed level in MAG. On the contrary, *cyp1a1* showed higher expressed level in MG (Table 2).

Additionally, in the after gonad differentiation comparison group (FAG vs. MAG), *bmp15*, *gdf9* and *org* were activated in ovary and *sox9a* was activated in testis. In sum up, there are 13 gonad development related DEGs were found from these comparison among different groups (Table 2).

### GO and KEGG enrichment analysis of sex-related DEGs

To better understand the possible functions involved in gonad development of yellow catfish, the DEGs from each comparison group were enriched with the GO database. As a result, several gonad development related genes were enriched into go terms. For example, *piwi* and *vasa* were enriched in germ plasm cellular part for MG vs. MAG comparison group (Additional files 2 and 3).

Correspondingly, the KEGG pathway enrichment analysis was performed. In BG vs. FG comparison group, *cyp1a1* was enriched in ovarian steroidogenesis pathway and *plk1* was enriched in cell cycle pathway. For FG vs. FGC group, *cyp1a1* was enriched in steroid hormone biosynthesis, *plk1* and *aurka* were enriched in oocyte meiosis. *Cyp1a1* was enriched in steroid hormone biosynthesis and ovarian steroidogenesis pathway for FG vs. FAG comparison group (Additional file 4).

Moreover, *cyp1a1* was enriched in steroid hormone biosynthesis pathway for BG vs. MG comparison group. *Aurka* was enriched in oocyte meiosis pathway for MG vs. MGC. *Cyp1a1* was also enriched in steroid hormone biosynthesis pathway for MG vs. MAG. *Bmp15*, *gdf9* and *org* were enriched in cytokine-cytokine receptor interaction pathway for FAG vs. MAG (Additional file 4).

### Time-course analysis of expression profiles of genes

We further clustered genes which exhibited the similar expression profiles in the entire gonad development stages of female and male respectively. Then, these clustered genes were annotated and enriched by GO and KEGG database (Additional files 5 and 6).

**Fig. 3:**
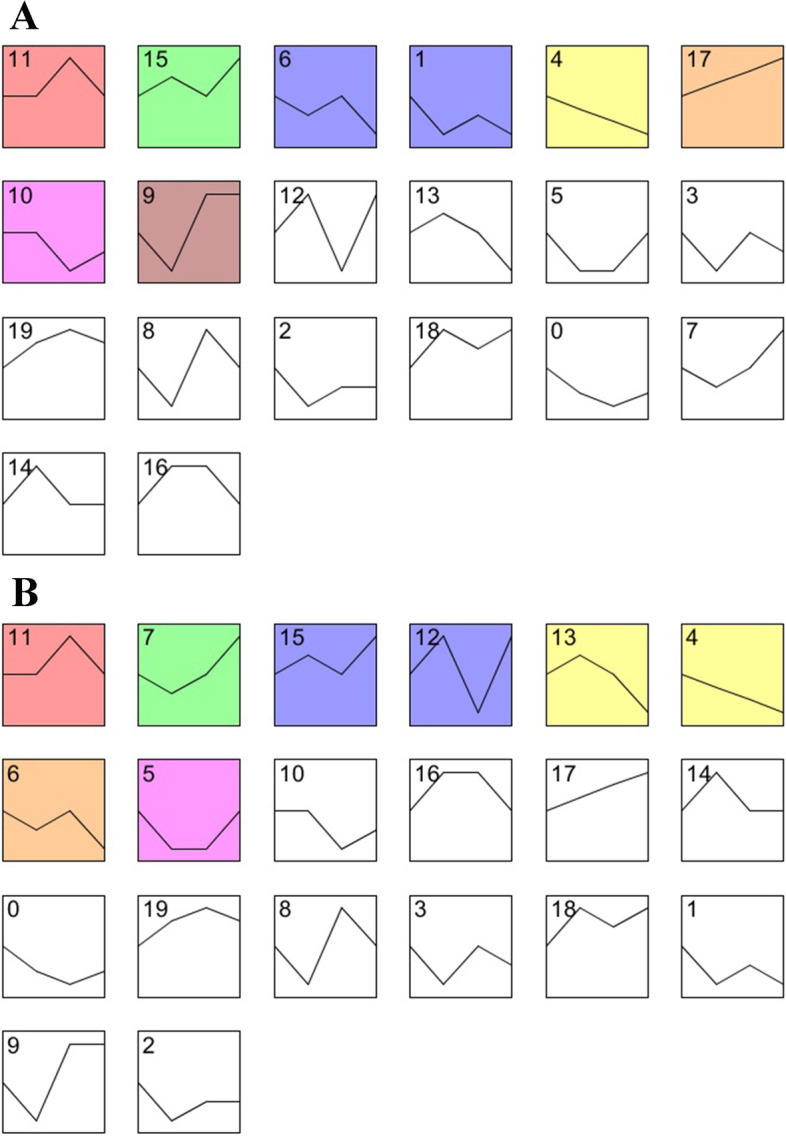
Gene clustering in different gonad development stages. Each box corresponds to a model expression profile. For each box, the left top number represents the profile number, the right bottom number represents the contained genes number. Colored profiles have a statistically significant number of gene assigned. **A** Expression pattern of female gonad development stages. **B** Expression pattern of male gonad development stages

For female, BG, FG, MGC and FAG were considered as four different ovary development stages. Seven clusters were obtained from these results (Fig. 3A). In profile 15, *aurka* was enriched in nuclear part (Additional files 5 and 7). In profile 17, *piwi* and *org* were enriched in intracellular; *vasa* was enriched in organelle and intracellular organelle (Additional files 5 and 7).

For male, BG, FGC, MG and MAG were considered as four different gonad development stages. Six clusters were obtained from these time-course transcriptome analysis results (Fig. 3B). In profile 7, *spata22* (spermatogenesis-associated protein 22), *tex11* (testis-expressed sequence 11 protein) and *tex15* (testis-expressed sequence 15 protein) were enriched in meiotic cell cycle and reproduction biological process (Additional files 5 and 8).

### Verification of DEGs by RT-qPCR

Seven DEGs were randomly selected for RT-qPCR validation to verify the RNA-Seq results of time-course gonadal transcriptome. The primers used for these genes are listed in Table 3.

**Table 3:** PCR primers used in this study

Genes	Sequence (5′-3′)	Product size (bp)	Gene_id
*amhr2*	GGCGAGGACGGTTTTCA	222	XLOC_006098
	AAGAGGACAGGCGTTGGTT		
*aurka*	CAAACCCCAGCCAGCAC	199	XLOC_019110
	GCTTCCAAACTTTCCCTTCC		
*bmp15*	ACTTCTCACGCTGGCTCAA	151	XLOC_000571
	CCCAACCAAGATCACGGA		
*cyp1a1*	CGCTCATTCGCAAACATC	271	XLOC_007208
	GGTTTCCGCTGCCCAC		
*gdf9*	GGGAGAAATCCATTCCAACC	192	XLOC_003614
	CGGAAGTCATAGAGGTCGC		
*plk1*	TAAGATTGGTGACTTCGGTTTG	251	XLOC_013011
	TGTGCCTGGGAATGGTGTA		
*vasa*	TTGCCAAATCAGGATACGAA	300	XLOC_017737
	TGCCACCATAAACCACGA		

**Fig. 4:**
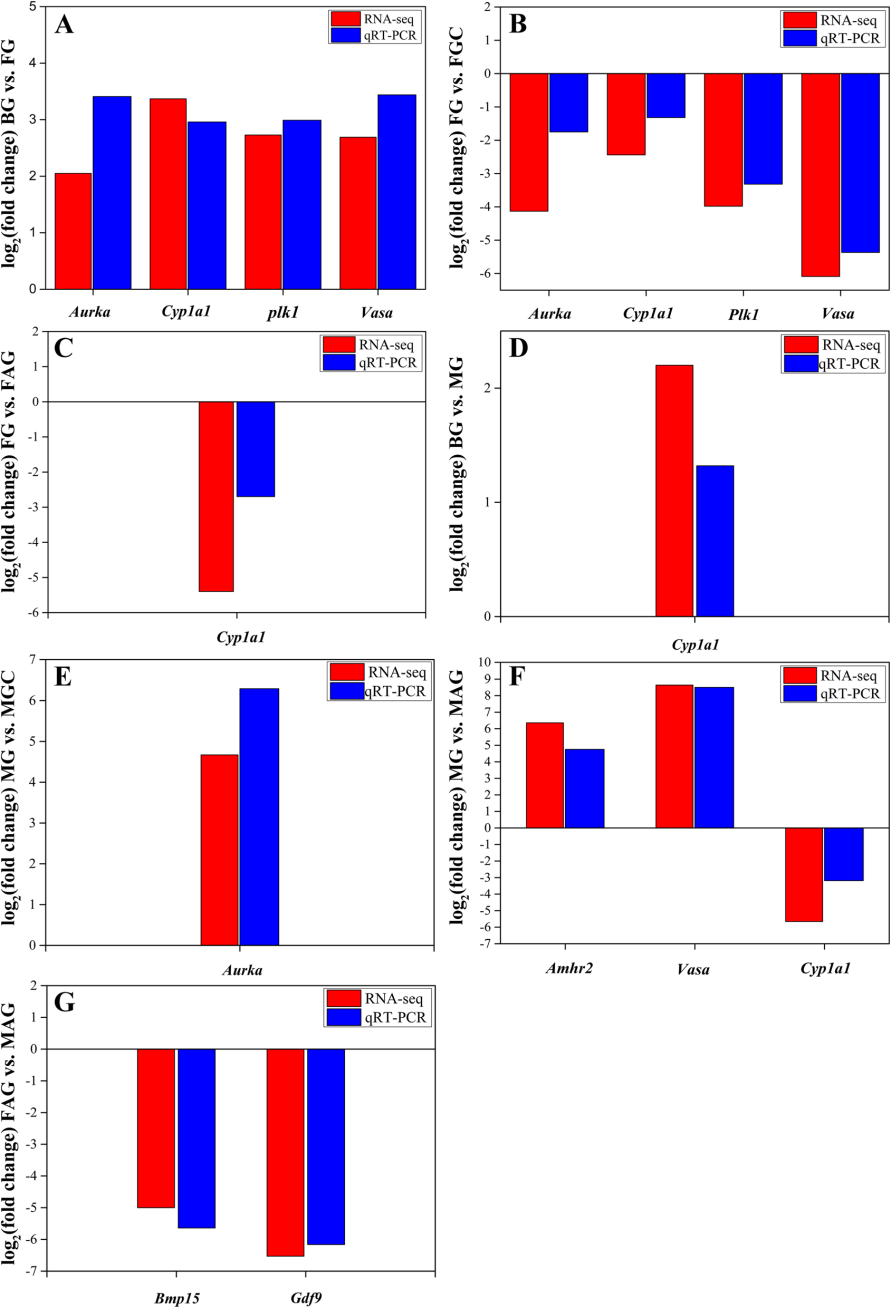
Validation of the expression patterns both in RNA-Seq (red) and RT-qPCR (blue). **A** BG vs. FG, **(B)** FG vs. FGC, **(C)** FG vs. FAG, **(D)** BG vs. MG, **(E)** MG vs. MGC, **(F)** MG vs. MAG, **(G)** FAG vs. MAG. Gene relative expression: log_2_(fold change). RT-qPCR fold changes is normalized by changes in beta-actin values. The averages of three relative quantities of biological replications were used in a two-tailed student’s t test with a 95% confidence level (*P* < 0.05) to determine the gene expression significance

The expression profiles of these selected genes obtained from RT-qPCR corresponded to the RNA-Seq results (Fig. 4).

## Discussion

In this work, a time-course gonadal transcriptome of yellow catfish was constructed with Illumina HiSeq 2000 sequencing technology. These results provided the valuable genetic resources to understand the mechanisms of gonad development and sexual dimorphism. A total of 2696 DEGs were obtained. Then, these DEGs were annotated and enriched by GO and KEGG databases. Time-course analysis were employed to detect the genes with similar expression profiles in female and male entire gonad development stages. Finally, 13 gonad development related genes were obtained from different gonad development stages of female and male yellow catfish (Table 2).

### *Cyp1a1* gene

Cyp1a1*,* a cytochrome P450 monooxygenase, involved in the metabolism of various endogenous substrates, including fatty acids, steroid hormones and vitamins [8–11]. Exhibits high catalytic activity for the formation of hydroxyestrogens from estrone (E1) and 17β-estradiol (E2) (Additional file 9). In this study, compare with BG, *cyp1a1* was up-regulated in early gonad differentiation stage (Additional file 9). Then, this gene was enriched in ovarian steroidogenesis (for female) and steroid hormone biosynthesis pathway (for male). These results suggested that *cyp1a1* may play a role in early gonad development through metabolizes steroid sex hormones (E1 and E2) in the gonad, and finally induces spermatogenesis and oogenesis.

### *Piwi* and *vasa* genes

Additionally, the time-specific expression patterns of *piwi* and *vasa* were detected in BG vs. FG and MG vs. MAG comparison group. In details, the two gonad development related genes showed highly expression levels in the period of ovary differentiation (in FG) and testis after differentiation (in MAG) respectively. *Piwi* controls the number of primordial germ cells (PGCs) via protecting maternal mRNA from decay and adult germ stem cell division in Drosophila [12]. In zebrafish, *piwi* is required for maintaining germ cells, because loss of *piwi* leads to germ cell loss by apoptosis [13]. For medaka, *piwi* is required not only for determining the PGC number but also for controlling PGC migration [14]. *Vasa* (ATP-dependent RNA helicase DDX4) gene is a RNA helicase of the DEAD-box helicase family, which plays a crucial role in germ cell formation. *Vasa* is localized specifically in germline cells, indicating a particular function in gonads [15]. Differential expression of a *vasa* homolog in the gonads of tilapia (*Oreochromis niloticus*) during gametogenesis suggests a possible role for *vasa* in regulating the meiotic progression of male and female germ cells [16]. So, time-specific expression patterns of *piwi* and *vasa* might be suggested that these two genes played more important roles in both ovary differentiation and testis maintain.

### Female-biased genes

**Fig. 5:**
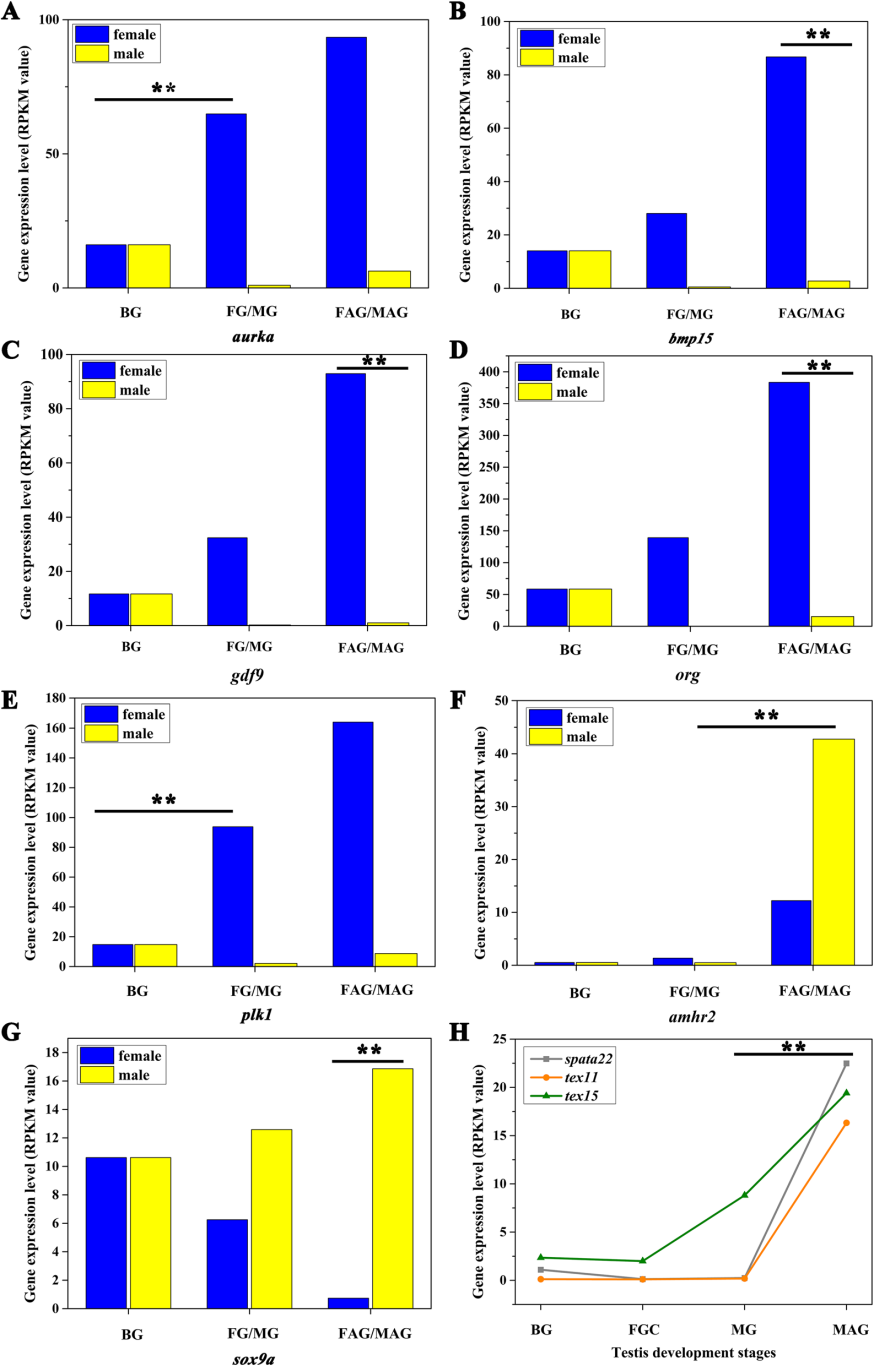
Sexual biased genes and clustered sex-related genes were identified from this study. **A-E** These histograms showed the female-biased sex-related genes. **F-G** These two male-biased sex-related genes were displayed by histograms. **H** *spata22*, *tex11* and *tex15* show increasing expression from FGC to MAG

According to our results, *aurka* was activated in early stage of ovary differentiation (Fig. 5A). Aurka, which is a centrosome-localized serine/threonine kinase crucial for cell cycle control, plays an important role in mitosis [17] and also regulates the meiotic cell cycle of Xenopus oocytes [18]. Meanwhile, *plk1*, which could be activated by *aukra*, was also up-regulated in FG (Fig. 5E). It has multiple regulatory roles in the cell cycle, including the control of cell cycle progression into mitosis [19]. These two genes were enriched in oocyte meiosis suggesting that *aurka* and *plk1* may play roles in proliferation and differentiation during the early stage of ovary development.

In FAG vs. MAG comparison group, *bmp15* and *gdf9* displayed the significant highly expression only in FAG (Fig. 5B and C). *Bmp15* and *gdf9* are two closely related members of the TGF-β superfamily who can regulate granulosa cell development [20–24]. Specifically, zebrafish *bmp15* mutants can initially develop as females. But during the juvenile stage, their sex revert to fertile males [21]. In our results, *bmp15* and *gdf9* had significantly higher expression levels in female after gonad differentiation. It is suggested that *bmp15* and *gdf9* may perform functions in maintaining the sexual phenotype of female yellow catfish.

### Male-biased genes

*Amhr2* and *sox9a* displayed the significant male-biased expression in male yellow catfish testis and they were up-regulated in MAG (Fig. 5F-G). Amh (anti-Muellerian hormone) is the gonadal hormone responsible for the regression of Mullerian ducts in male fetuses during mammalian embryogenesis [25, 26]. Amh signals through the *amhr2* to regulate the differentiation and growth of target cells in mammals [27]. In fish, an *amhr2* mutation results in a male-to-female sex change in XY medaka suggested that *amhr2* is involved in sex determination [28]. Our laboratory have observed highly expression of *sox9a* in 1-year-old and 2-year-old yellow catfish testis. The present results consist with our previous studies which indicated that these two genes are not only important to testis differentiation but also are crucial to the maintenance and development of testis.

### The clustered genes

Additionally, from the results of STEM, we found that *spata22*, *tex11* and *tex15* showed increased expressions from FGC to MAG (Fig. 5H). *Spata22* is required early in meiotic prophase in both male and female germ cells [29]. *Tex11* and *tex15* display testis-specific expression which is required for meiotic recombination [30, 31].

## Conclusions

Thanks for published high quality chromosome-level yellow catfish reference genome, a time-course gonadal transcriptome analysis was performed with cufflinks for yellow catfish to explore the mechanisms of gonad development and sexual dimorphism. A final assembly which consists of 41,329 transcripts was generated and annotated. GO and KEGG enrichment analysis and STEM cluster analysis revealed that all of 13 gonad development related genes were enriched in multiple go terms and pathways including meiotic cell cycle, reproduction biological process, ovarian steroidogenesis, steroid hormone biosynthesis, and oocyte meiosis pathway. These gonad development related genes showed various expression patterns. For example, *cyp1a1* was up-regulated in gonad differentiation stages and enriched in ovarian steroidogenesis (in FG) or steroid hormone biosynthesis pathway (in MG). These results indicated that *cyp1a1* may play a role in early gonad development through metabolizes steroid sex hormones (E1 and E2) in the gonad, and finally induces spermatogenesis and oogenesis. Moreover, *piwi* and *vasa* showed highly expression levels in ovary differentiation stage (FG) and after testis differentiation stage (MAG) respectively. It is suggested that these two genes may play an important role in ovary differentiation and testis maintaining. These results provided a reference for subsequent research on the mechanism of sexual dimorphism and sex control of yellow catfish.

## Materials and methods

### Samples and RNA extraction

Depended on previous studies, in yellow catfish, the gonad differentiation was observed at 13dph (day post-hatch) and 54 dph in females and males respectively [32, 33]. To compare the gene expression patterns of different gonad stages, a total of 42 full-sibling offsprings were sampled at before gonad differentiation (3dph), gonad differentiation (13dph for female and 54dph for male), and after gonad differentiation (90dph) respectively. Additionally, at female gonad differentiation stage, male individuals were collected into a control group (FGC). Similarly, at male gonad differentiation stage, female individuals were collected into a control group (MGC). All details of sampled groups were listed in Table 4.

**Table 4:** Summary of sampling and grouping from different gonad development stages

Group	Gonad differentiation Stage	Individuals number	Gender of individuals
BG	before gonad differentiation	6	undifferentiated
FG	female gonad differentiation	6	female
FGC	female gonad differentiation	6	male
MG	male gonad differentiation	6	male
MGC	male gonad differentiation	6	female
FAG	after gonad differentiation	6	female
MAG	after gonad differentiation	6	male

The sex of each individual was confirmed by sex-linked marker as described previously [34]. Then, for each group, gonad tissues were sampled and pooled from 6 individuals to obtain enough total RNA and avoid individual variations. Total RNAs were extracted by Trizol method with manufacturer’s protocol (Ambion). The concentration and quality of each RNA sample were examined using a NanoDrop-2000 spectrophotometer (Thermo Scientific, Waltham, MA, USA). The RNA integrity was checked by ethidium bromide staining of 28S and 18S ribosomal bands on a 1% agarose gel.

### Libraries construction and sequencing

Equal amounts of high-quality RNA samples were used to synthesize cDNA libraries. Briefly, mRNAs were purified from total RNAs and used as templates to synthesize the first-strand and the second-strand of cDNAs, according to the protocol of Super Script Double-Stranded cDNA Synthesis kit (Thermo Fisher Scientific, MA, USA). cDNAs were cut into short fragments following the TruSeq RNA sample preparation guide. After end repair and the addition of poly (A), the short fragments were ligated with sequencing adapters and enriched by PCR amplification to construct the cDNA library templates. Finally, transcriptome sequencing was performed using the Illumina HiSeq 2000 platform, which generated about 100-bp paired-end (PE) raw reads.

### Sequence assembly and annotation

Raw reads were cleaned by removing the adaptor-containing sequences and low-quality reads. All clean reads were aligned and mapped using Tophat [35] to the published yellow catfish reference genome [36]. These mapped reads were subsequently used by Cufflinks [37] to generate non-redundant set of transcript contigs representing the transcriptome of yellow catfish. Cuffnorm was run on each group reads separately to estimate transcript abundance (read counts). Cuffdiff was then performed to differential expression analysis of 1) BG vs. FG; 2) BG vs. MG; 3) FG vs. FGC; 4) MG vs. MGC; 5) FG vs. FAG; 6) MG vs. MAG; and 7) FAG vs. MAG respectively. The considered thresholds of DEGs including *p*-value ≦ 0.05 and |log_2_ (fold change)| ≥ 1.

All assembled transcripts were annotated using several popular publicly available databases, including NCBI non-redundant protein sequences (Nr), Gene Ontology (GO) database [38] and Kyoto Encyclopedia of Genes and Genomes (KEGG) database [39] by using BLASTX [40] with a cut-off of *E* ≤ 1e-5, OmicsX, and KOBAS respectively. Combined with the results of annotation and enrichment of DEGs, two criteria were used to identify the gonad development related DEGs in yellow catfish: 1) the DEGs were enriched (p-value ≤0.05) in gonad related GO terms or KEGG pathways, such as ovarian steroidogenesis, reproduction biological process; 2) the DEGs were enriched in GO terms or KEGG pathways and reported by previous gonad development studies, such as *gdf9 and vasa*.

### Gene expression during the developmental stages of gonad

Depended on the transcript abundance results produced by Cuffnorm, Short Time-series Expression Miner (STEM) [41] were employed to cluster these genes which exhibited the similar expression profiles in entire gonad development stages (from before gonad differentiation to after gonad differentiation stage) in female and male respectively. Genes were considered as having similar expression patterns which were clustered into a same cluster. The clustered profile with FDR value ≤0.05 were regarded as a valid cluster.

### Real-time PCR verification

Seven randomly selected DEGs were validated by real-time quantitative PCR (RT-qPCR) to prove the reliability of RNA-seq results. The PrimerScript TM RT regent Kit with gDNA Eraser was used to cDNA synthesis following the manufacturer’s instructions. The primer information for these genes are available in Table 3. *β-actin* was employed as the endogenous reference gene. The qPCR mixture consisted of 10 μl of 2 × Master Mix, 1 μl of cDNA, 0.5 μl of each primer and 8 μl of ddH_2_O under the following conditions to collect fluorescence: denaturation at 95 °C for 30 min, followed by 40 cycles of amplification at 95 °C for 5 s and 60 °C for 40s. To establish the melting curve of the PCR product, the reaction was performed at 95 °C for 10s, 60 °C for 60s, and 95 °C for 15 s, after which it was slowly heated from 60 °C to 99 °C. The relative expression levels of target genes in each sample were individually determined using the 2^-∆∆CT^ method [42].

## Supplementary Information

**Additional file 1: Table S1.** Nr, GO and KEGG annotation results of the transcriptome.

**Additional file 2: Fig. S1.** Bubble plot for the GO enrichment results of DEGs.

**Additional file 3: Table S2.** GO enrichment results of the DEGs.

**Additional file 4: Table S3.** KEGG enrichment results of the DEGs.

**Additional file 5: Table S4.** GO enrichment results of the clustered genes which exhibit the similar expression profiles in female or male entire gonad development stages.

**Additional file 6: Table S5.** KEGG enrichment results of the clustered genes which exhibit the similar expression profiles in female or male entire gonad development stages.

**Additional file 7: Table S6.** Bubble plot for the GO enrichment results of gene clusters in female gonad development stages.

**Additional file 8: Fig. S2.** Bubble plot for the GO enrichment results of gene clusters in male gonad development stages.

**Additional file 9: Fig. S3.** Steroid hormone biosynthesis pathway. The heat map shows the expression of cyp1a1 (log2 (FPKM)) in different gonad development stages.

## Data Availability

All datasets from the Illumina sequencing platform can be found in the Short Read Archive (SRA) database of the National Center for Biotechnology Information (NCBI) under accession number (PRJNA668564).

## Abbreviations

BG: Before gonad differentiation
BUSCO: Benchmarking Universal Single-Copy Orthologs
DEG: Different expression genes
FAG: Female after gonad differentiation
FDR: False discovery rate
FG: Female gonad differentiation
FGC: Female gonad differentiation control
GO: Gene ontology
KEGG: Kyoto Encyclopedia of Genes and Genomes
MAG: Male after gonad differentiation
MG: Male gonad differentiation
MGC: Male gonad differentiation control
Nr: Non-redundant protein sequences
PCR: Polymerase chain reaction
RT-qPCR: Real-time quantitative PCR
STEM: Short time-series expression miner
