# Supplementary figures and images for: A time-course transcriptome analysis of gonads from yellow catfish (Pelteobagrus fulvidraco) reveals genes associated with gonad development
Source: BMC Genomics. 2022 May 31;23(Suppl 1):409. doi: 10.1186/s12864-022-08651-0 (PMC9153201; doi:10.1186/s12864-022-08651-0)

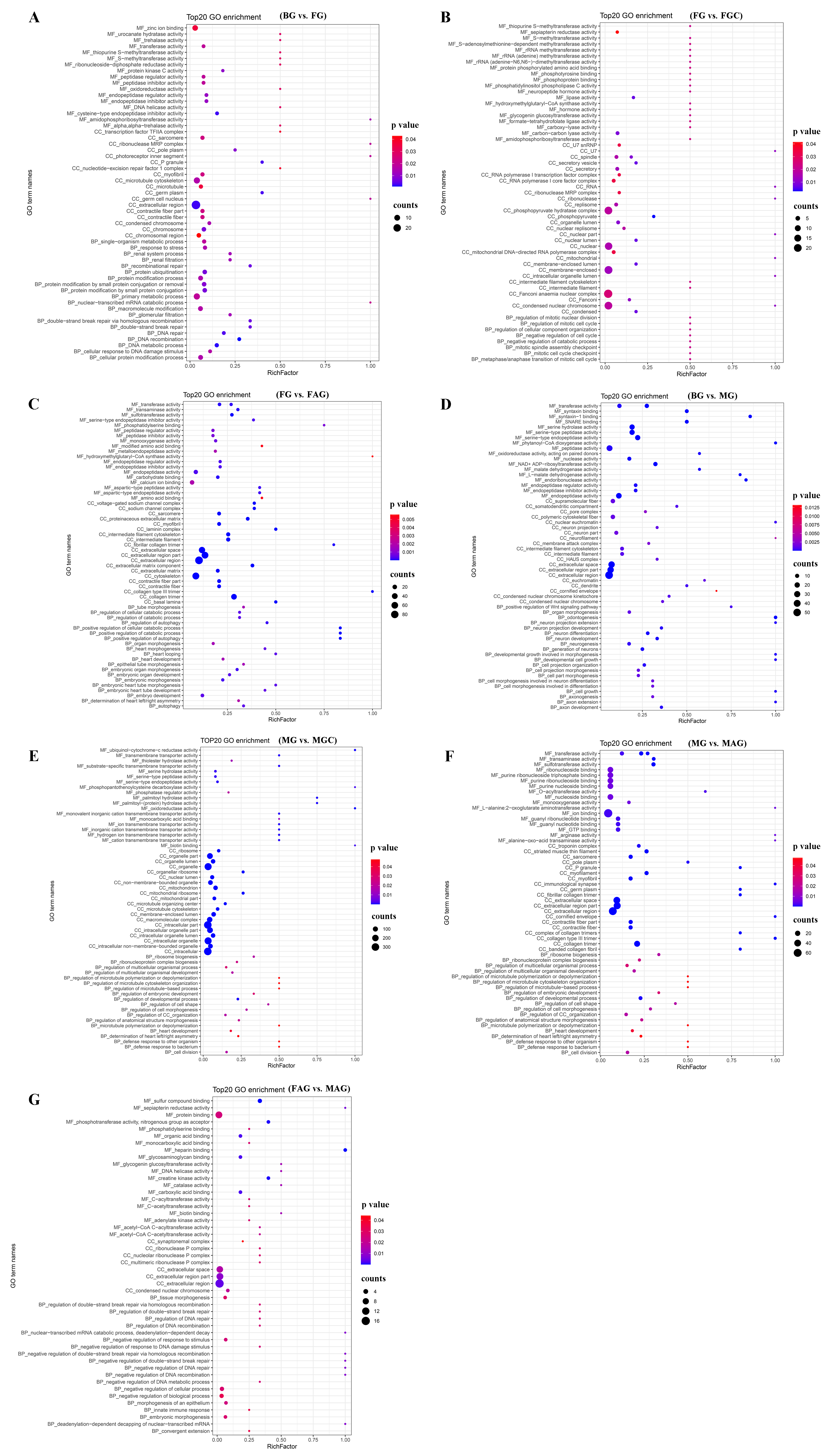

Supplement: Supplementary file 2 — Additional file 2: Fig. S1. Bubble plot for the GO enrichment results of DEGs. [file 12864_2022_8651_MOESM2_ESM.png]

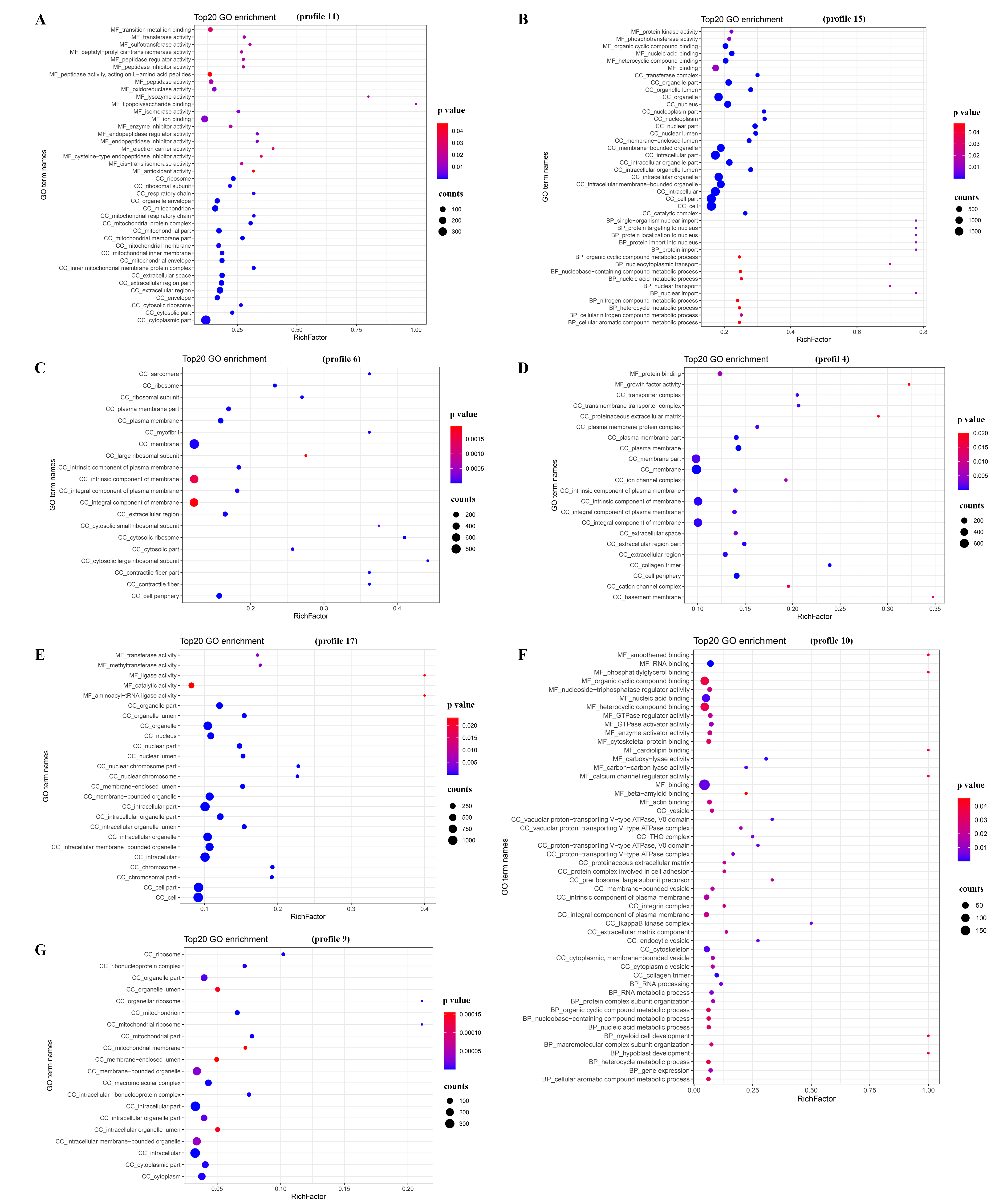

Supplement: Supplementary file 7 — Additional file 7: Table S6. Bubble plot for the GO enrichment results of gene clusters in female gonad development stages. [file 12864_2022_8651_MOESM7_ESM.png]

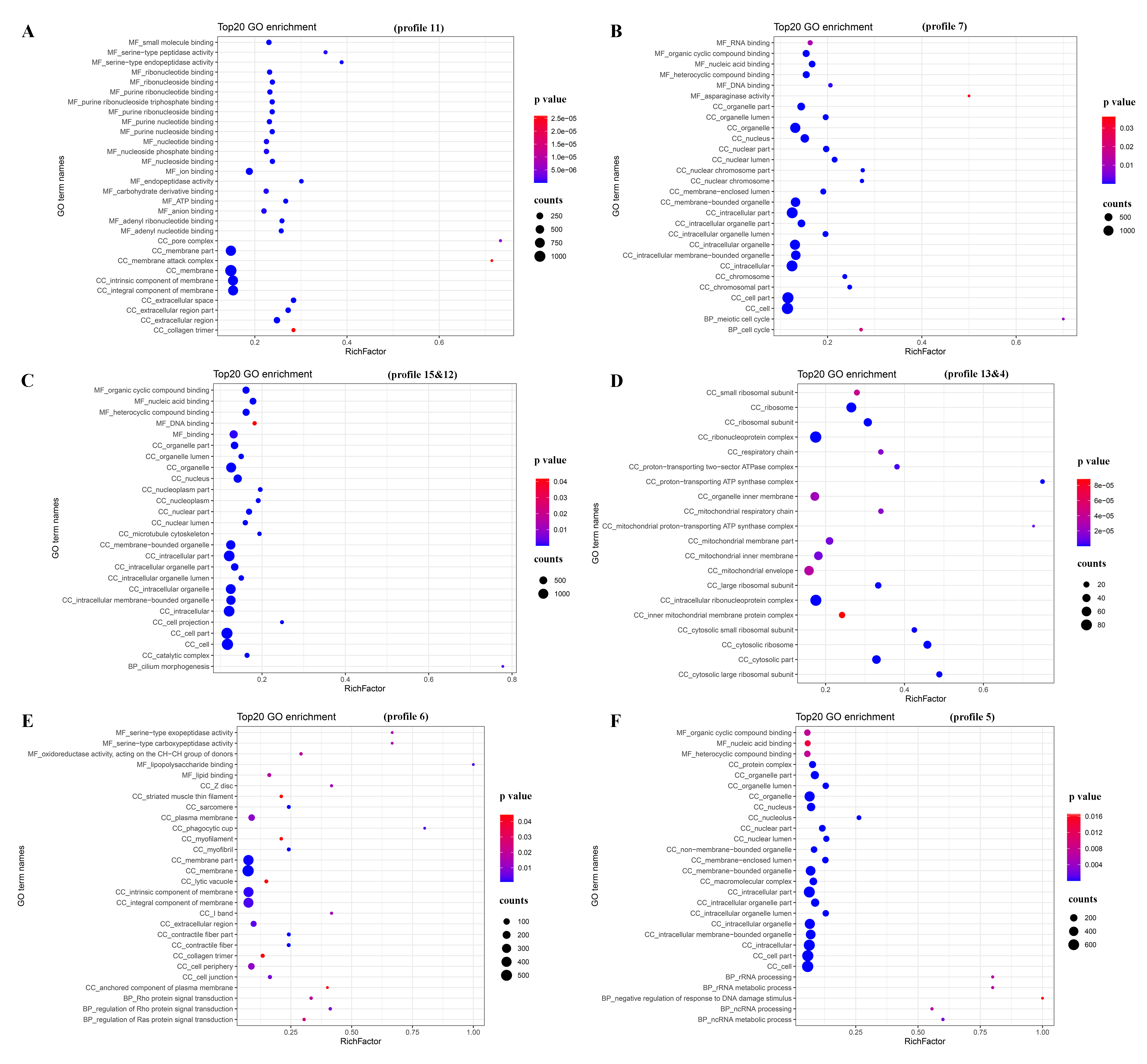

Supplement: Supplementary file 8 — Additional file 8: Fig. S2. Bubble plot for the GO enrichment results of gene clusters in male gonad development stages. [file 12864_2022_8651_MOESM8_ESM.png]

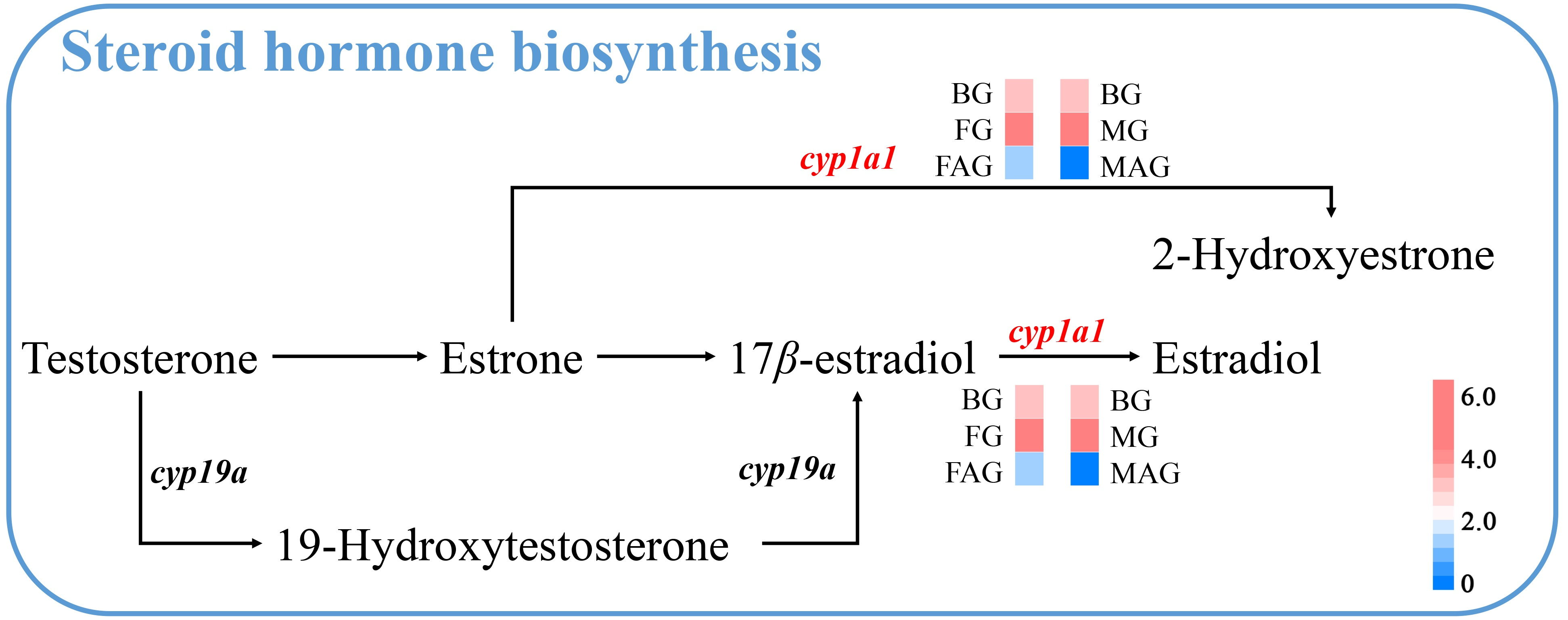

Supplement: Supplementary file 9 — Additional file 9: Fig. S3. Steroid hormone biosynthesis pathway. The heat map shows the expression of cyp1a1 (log2 (FPKM)) in different gonad development stages. [file 12864_2022_8651_MOESM9_ESM.png]
